# Phosphorylation of Spastin Promotes the Surface Delivery and Synaptic Function of AMPA Receptors

**DOI:** 10.3389/fncel.2022.809934

**Published:** 2022-03-28

**Authors:** Li Chen, Hanjie Wang, Shuhan Cha, Jiong Li, Jiaqi Zhang, Jiaming Wu, Guoqing Guo, Jifeng Zhang

**Affiliations:** ^1^Department of Anatomy, Neuroscience Laboratory for Cognitive and Developmental Disorders, Medical College of Jinan University, Guangzhou, China; ^2^Department of Neurosurgery, The First Affiliated Hospital of Jinan University, Guangzhou, China

**Keywords:** spastin, phosphorylation, AMPA receptor, synaptic plasticity, microtubule

## Abstract

Synaptic plasticity is essential for cognitive functions such as learning and memory. One of the mechanisms involved in synaptic plasticity is the dynamic delivery of AMPA receptors (AMPARs) in and out of synapses. Mutations of *SPAST*, which encodes SPASTIN, a microtubule-severing protein, are considered the most common cause of hereditary spastic paraparesis (HSP). In some cases, patients with HSP also manifest cognitive impairment. In addition, mice with Spastin depletion exhibit working and associative memory deficits and reduced AMPAR levels. However, the exact effect and molecular mechanism of Spastin on AMPARs trafficking has remained unclear. Here, we report that Spastin interacts with AMPAR, and phosphorylation of Spastin enhances its interaction with AMPAR subunit GluA2. Further study shows that phosphorylation of Spastin can increase AMPAR GluA2 surface expression and the amplitude and frequency of miniature excitatory synaptic currents (mEPSC) in cultured hippocampal neurons. Moreover, phosphorylation of Spastin at Ser210 is crucial for GluA2 surface expression. Phosphorylation of Spastin K353A, which obliterates microtubule-severing activity, also promotes AMPAR GluA2 subunit trafficking to the surface and increases the amplitude and frequency of mEPSCs in cultured neurons. Taken together, our data demonstrate that Spastin phosphorylation promotes the surface delivery of the AMPAR GluA2 subunit independent of microtubule dynamics.

## Introduction

AMPA-type glutamate receptors (AMPARs) are heterotetrameric assemblies of four highly homologous subunits, GluA1–4, that are highly enriched at synapses (Herguedas et al., 2016; Diering and Huganir, 2018). In the central nervous system, postsynaptic AMPARs mediate the majority of fast excitatory transmission. Interestingly, AMPARs are not static components at synapses, rather, they are continuously being delivered and removed in and out of the synapses (Moretto and Passafaro, 2018). The dynamic trafficking of AMPARs into and out of the synaptic membrane is crucial for synaptic plasticity, which is thought to be one of the key cellular mechanisms underlying cognitive functions such as learning and memory (Anggono and Huganir, 2012; Henley and Wilkinson, 2013). Generally speaking, an increased number of synaptic AMPARs leads to long-term potentiation (LTP), which promotes learning and memory, whereas the removal of surface AMPARs results in long-term depression (LTD; Anggono and Huganir, 2012; Henley and Wilkinson, 2013). This dynamic behavior of AMPAR involves a complex protein-protein interaction network, from receptor biosynthesis to their transport along dendrites and finally insertion and removal from the postsynaptic membrane (Greger and Esteban, 2007; Kneussel and Hausrat, 2016). Thus, elucidating how proteins regulate the trafficking of AMPARs is critical for our understanding of synaptic plasticity and human cognitive behavior.

Hereditary spastic paraplegia (HSP) is a heterogeneous group of genetic neurodegenerative disorders, characterized by distinct lower limb spasticity and weakness (Novarino et al., 2014; Walusinski, 2020). To date, there are about 100 loci/88 spastic paraplegia genes (SPG) involved in the pathogenesis of HSP (Elsayed et al., 2021). More than 40% of HSP cases originate from mutations in the *SPG4* gene, which encodes SPASTIN, a microtubule-severing protein (Kara et al., 2016; Erfanian Omidvar et al., 2021). Spastin is widely expressed in the spinal cord and brain (Solowska et al., 2008, 2010). In the central nervous system, it is mainly distributed in regions such as the cerebral cortex, cerebellum, hippocampus, amygdala, substantia nigra, and striatum (Solowska et al., 2008). The classic function of Spastin is to sever long microtubules into a number of short segments which mainly relies on the microtubule-binding domain (MTBD) and AAA ATPase catalytic domain (Blackstone et al., 2011). The MTBD is responsible for binding tubulin in an ATP-independent manner and the AAA domain forms a circular hexamer with a central pore. It is proposed that the C-terminal tail of tubulin is pulled into this hexamer, generating a mechanical force that breaks the microtubules (Salinas et al., 2005; Roll-Mecak and Vale, 2008). As HSP is a motor neuron disease caused by a progressive degeneration of the motor axons of the corticospinal tract (Salinas et al., 2008), numerous studies have focused on the causative mechanism of motor axon injuries of the corticospinal tract by Spastin. These studies have shown that the severing ability of Spastin has a crucial role in axon growth and axonal transport (Yu et al., 2008; Kasher et al., 2009; Stone et al., 2012; Fassier et al., 2013). Thus, impairment of axonal growth and transport caused by insufficient microtubule cleavage is considered to be one of the reasons for the pyramidal syndrome described in SPG4-linked HSP (Solowska and Baas, 2015).

Originally, SPG4-linked HSP had been considered as a pure form (in which only a pyramidal syndrome is found), however, later studies reported that patients with SPG4-HSP also exhibit cognitive impairment (Orlacchio et al., 2004; Murphy et al., 2009; Chelban et al., 2017; Akaba et al., 2021; Erfanian Omidvar et al., 2021; Giordani et al., 2021). In addition, mice with Spastin depletion exhibit working and associative memory deficits and reduced AMPA receptor levels (Lopes et al., 2020). One of the mechanisms explaining the influence of synaptic plasticity on cognitive behavior is AMPAR trafficking (Forrest et al., 2018); thus, further exploration of how Spastin regulates AMPAR trafficking is warranted. Our study of cultured hippocampal neurons showed that in addition to its influence on axon outgrowth, Spastin also promotes dendrite development (Ji et al., 2018). It interacts with collapsin response mediator proteins (CRMPs) to promote dendrite outgrowth and branch formation (Ji et al., 2018; Li et al., 2021). Moreover, phosphorylation of Spastin was found to play a key role during this process (Li et al., 2021). Phosphorylation, as the most common and important posttranslational modification of proteins, has critical and well-known functions in diverse cellular processes. Although a recent study has shown that Spastin depletion reduces AMPA receptor (AMPAR) levels (Lopes et al., 2020), the role of Spastin and its phosphorylation on AMPA receptor trafficking is not fully understood.

In this study, we reported that Spastin interacts with all four subunits of AMPA receptors. Then, using phosphorylation site mutations, we found that phosphorylation of Spastin enhances its interaction with AMPAR subunit GluA2. Further investigation showed that overexpression of phosphorylated Spastin mutation increases the surface expression of AMPAR levels. Meanwhile, the synaptic function was also increased. Moreover, our results identified Ser210 as the key phosphorylation site of Spastin involved in AMPAR trafficking. Finally, by phosphorylation of Spastin K353A, which obliterates microtubule-severing activity, we clarified that microtubule motility was not involved in Spastin phosphorylation-mediated AMPAR trafficking. Taken together, our data provide new and important insights into the role of Spastin in AMPAR trafficking and advances our understanding of the synaptic plasticity and cognitive dysfunction in HSP.

## Materials and Methods

### Animals

The experiments were undertaken with 1-month-old and 1-day-old specific pathogen-free Sprague Dawley (SD) rats purchased from the Experimental Animal Center of Sun Yat-sen University. We conducted the animal experiments in strict accordance with the recommendations in the Guide for the Care and Use of Laboratory Animals produced by the National Institutes of Health. The protocol was approved by the Institutional Animal Care and Use Committee at Jinan University, China. All efforts were taken to minimize the suffering and the number of animals used.

### Construction of Plasmids

Green fluorescent protein (GFP)-Spastin, mCherry-Spastin, and Glutathione-S-transferase (GST)-Spastin constructs were described previously (Cha et al., 2016; Ji et al., 2018). Point mutations S210A, S233A, T271A, S562A (mutation of serine and threonine to alanine to mimic dephosphorylated Spastin), S210D, S233D, T271D, S562D (mutation of serine and threonine to aspartic acid to mimic phosphorylated Spastin), K353A (mutation of lysine to alanine), and R464C (mutation of arginine to cysteine) were generated by using the Quickchange Kit (Agilent, Santa Clara, CA), and the positive clone was confirmed by sequencing.

### Hippocampal Neuron Culture and Transfection

Standard hippocampal neuron culture and transfection were performed as described in our previous study (Zhang et al., 2012). Briefly, hippocampi were extracted from 1-day-old SD rats. After hippocampi were cut into pieces, they were digested using 0.125% trypsin. Finally, rat hippocampal neurons were plated onto a poly-D-lysine coated glass coverslip at a density of 1 × 10^4^ cells/cm^2^. When cells were cultured for 13 days *in vitro* (DIV 13), different constructs were transfected into neurons using the calcium-phosphate method. All experiments were performed after transfection for 48 h.

### COS1 and HEK293T Cell Culture and Transfection

COS1 cells and HEK293T cells were cultured in a 5% CO_2_ incubator at 37°C. Transfection of the constructs was performed in 24-well plates or 10-cm dishes with Lipofectamine 2000 (Invitrogen, Waltham, MA). After transfection for 48 h, COS1 cells were fixed for performing fluorescence immunostaining. HEK293T cells were harvested for performing immunoprecipitation.

### GST Pull-Down Assay

The plasmids for GST-Spastin and its mutants were transformed into the BL21 strain of* E. coli* (Invitrogen). The GST-fusion proteins expression was performed as described previously (Ji et al., 2018). Approximately, 400 μg brain protein from 1-month-old SD rats was incubated with 5 μg GST-fusion protein under gentle rotation at 4°C overnight. Then the binding proteins were eluted and analyzed using western blotting.

### Co-immunoprecipitation Assay

HEK293T cells were co-transfected with Flag-GluA2 and GFP, GFP-Spastin, and its phosphomimetic and dephosphomimetic mutants. The co-IP assay was performed after transfection for 48 h, as per a previously described method (Cheng et al., 2022). HEK293T cells were lysed with a cold immunoprecipitation (IP) lysis buffer (Beyotime, Shanghai, China; 25 mM Tris-Cl pH 7.4, 100 mM NaCl, 1 mM ethylenediaminetetraacetic acid, 0.5% NP40) supplemented with protease inhibitor cocktail (Roche, Basel, Switzerland) for 30 min. The lysates were harvested and centrifuged at 12,000 rpm at 4°C for 20 min. Cell extracts were determined using the bicinchoninic acid assay (BCA) and incubated with anti-GFP agarose beads (KT Health, Shenzhen, China) at 4°C for 3 h. Following this, the beads were collected and washed twice with IP lysis buffer and once with IP wash buffer with 0.05% NP-40 in PBS. The immune complexes were collected and eluted six times with IP wash buffer. The samples were analyzed by Western blotting using anti-Flag and anti-GFP antibodies.

### Fluorescence Immunostaining

Immunofluorescence staining was performed after cells were transfected for 48 h, as per a previously described method (Zhang et al., 2012; Cheng et al., 2022). For tubulin immunofluorescence staining, COS1 cells were permeabilized by 0.1% (v/v) Triton X-100 dissolved in tris-buffered saline. The primary antibody targeting tubulin (Abcam, Cambridge, United Kingdom) was used at dilution 1:500 and the secondary antibody Alexa Fluor 555 (Life Technologies, Carlsbad, CA) was diluted at 1:1,000. For surface GluA2 immunofluorescence staining, hippocampal neurons and HEK293T cells expressing GluA2 subunits were not permeabilized. The primary antibodies targeting GluA2 (Millipore, Burlington, MA) were used at 1:200 dilutions. The secondary antibodies Alexa Fluor 647 and Alexa Fluor 555 (Life Technologies, Carlsbad, CA) were diluted to 1:800. After staining, images were randomly captured in a blinded manner under a Carl Zeiss LSM 700 confocal microscope (Zeiss, Oberkochen, Germany) with a 63× oil microscope. Fluorescence-integrated density measurements were made using ImageJ software (version ImageJ 2.x; NIH). Briefly, three dendritic regions of ~25 μm for each neuron were randomly selected and their average was obtained. Surface GluA2 was calculated by dividing the intensity corresponding to the transfected cells by the values corresponding to the non-transfected cells. Twenty neurons were counted from three independent experiments.

### Electrophysiology

After culturing hippocampal neurons for 13 DIV, they were transfected with GFP, GFP-Spastin, and other constructs. After transfection for 48 h, electrophysiology assays were performed as per a previously described method (Zhang et al., 2020). Miniature excitatory synaptic currents (mEPSCs) were obtained using whole-cell patch-clamp recordings at 20°C–22°C. During recordings, cultured hippocampal neurons were bathed in an extracellular solution (in mM): 128 NaCl, 5 KCl, 1 MgCl_2_, 2 CaCl_2_, 20 HEPES, 15 glucose, 1 tetrodotoxin, and 100 μM picrotoxin. The intracellular solution contained the following (in mM): 147 KCl, 5 Na_2_-phosphocreatine, 2 EGTA, 10 HEPES, 2 MgATP and 0.3 Na_2_GTP. Recordings were performed in voltage clamp mode, at a holding potential of −70 mV, using a Multiclamp 700 B amplifier (Molecular Devices, San Jose, CA) and Clampex 10.5 software (Axon Instruments, Union City, CA). Series resistance below 30 MΩ was monitored during recordings. Signals were sampled at 10 kHz, filtered at 1 kHz and MiniAnalysis software was used for analyzing signals (Synaptosoft, Inc., Fort Lee, NJ).

### Statistical Analysis

Statistical analysis was performed with GraphPad Prism 5 (GraphPad Software, San Diego, CA). All data are presented as the mean ± SEM. One-way ANOVA followed by Tukey’s *post-hoc* tests was used to determine differences among multiple groups. *P*-value < 0.05 was considered statistically significant.

## Results

### Phosphorylation of Spastin Increased Its Interaction With AMPAR GluA2 Subunit

**Figure 1:**
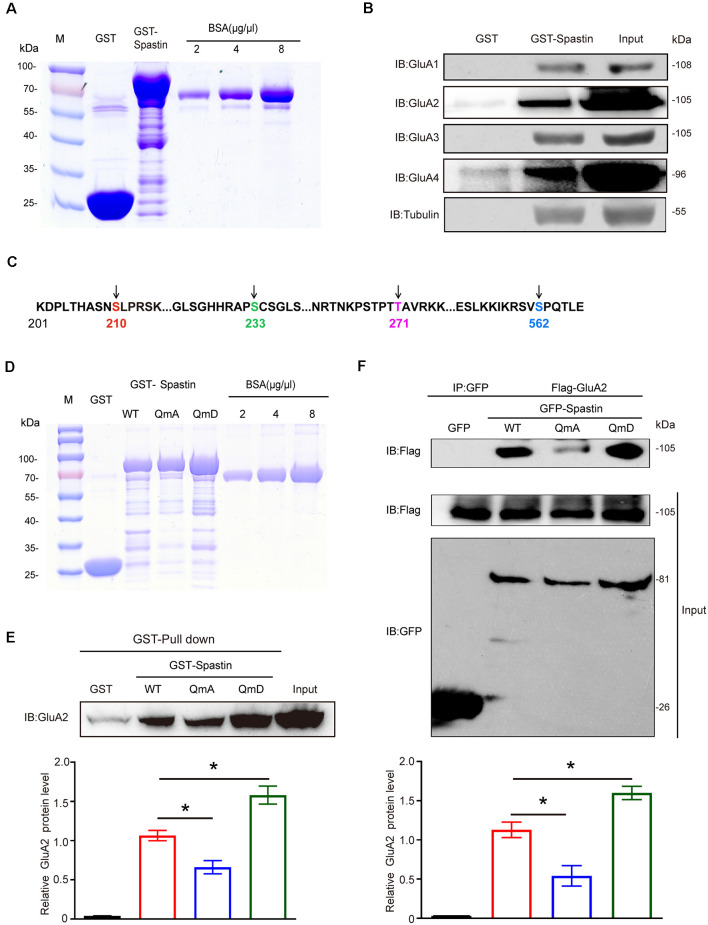
Spastin interacted with AMPARs and phosphorylation of Spastin increased its binding ability with GluA2. **(A)** Purified glutathione-S-transferase (GST), GST-Spastin proteins. **(B)** Pulldown assay was performed using GST-tagged Spastin or GST incubated with 1-month-old SD rat brain lysates. Immunoblotting of input and bound proteins was performed using antibodies against GluA1-A4 and tubulin. **(C)** Schematic of the four phosphorylation sites within the rat Spastin sequence. Numbers represent amino acid residues within the Spastin sequence. **(D)** Purified proteins of GST, GST-Spastin, and its phosphomimetic and dephosphomimetic mutants. **(E)** Pulldown assay was performed using GST, GST-tagged Spastin, and its mutants incubated with 1-month-old SD rat brain lysates. Immunoblotting of input and bound proteins was performed using antibodies against GluA2 (top); quantification of the relative binding of GluA2 to GST, GST-Spastin, and its mutants (bottom), *n* = 3 independent experiments, *p < 0.05 compared to GST-Spastin WT group. **(F)** HEK293T cells co-transfected with Flag-GluA2 and GFP, GFP-Spastin, and its mutants were lysed for co-IP assay. Immunoblotting of input and bound proteins was performed using antibodies against GFP and Flag respectively (top). Quantification of the relative binding of GluA2 to GFP, GFP-Spastin, and its mutants (bottom), *n* = 3 independent experiments, *p < 0.05 compared to GFP-Spastin WT group.

To investigate the relationships between Spastin and AMPAR, we first constructed GST-Spastin plasmid and purified the protein (Figure 1A). Then GST and GST-Spastin fusion protein was purified and incubated with the brain lysates of 1-month-old rats. GST pulldown analysis showed that Spastin interacted with all the four subunits (GluA1-GluA4) of AMPA receptors (Figure 1B). Simultaneously, to ensure the accuracy of the experimental system, we also performed a pulldown assay to examine the relationship between Spastin and tubulin as a positive control (Figure 1B). Since our previous study has shown that phosphorylation of Spastin can change its binding ability to interacted proteins, we speculated that phosphorylation of Spastin may also influence Spastin–AMPAR interaction. As a vast majority of AMPARs are GluA1/2 and GluA2/3 heteromers and most of the synaptic AMPARs within the brain contain GluA2 subunits (Bats et al., 2013), we selected GluA2 to evaluate whether Spastin phosphorylation affects the binding ability between Spastin and AMPAR. Large-scale mass spectrometry analyses have revealed four different potential phosphorylation sites in Spastin. These sites are S210, S233, T271, and S562 in rats (Figure 1C), corresponding to human S245, S268, T303, and S597, and are evolutionarily conserved in rats, humans, and mice^1^. With the exception of S268, which is reported to be phosphorylated by HIPK2, the upstream kinase at other sites is still unknown (Pisciottani et al., 2019). Therefore, in our study, we chose a genetic approach wherein we mutated all the phosphorylation sites (Ser210, Ser233, Thr271, and Ser562) of Spastin and constructed a phosphomimetic mutant (Spastin QmD) and dephosphomimetic mutant (Spastin QmA) of Spastin. In Spastin QmD, Ser210, Ser233, Thr271, and Ser562 were mutated to aspartic acid, whereas, in Spastin QmA, Ser210, Ser233, Thr271, and Ser562 were mutated to alanine. After the two mutants were successfully constructed, fusion proteins of GST, GST-Spastin WT, QmA, and QmD were purified (Figure 1D) for GST pulldown assays. As shown in Figure 1E, the binding ability of phosphomimetic mutant Spastin QmD with GluA2 was enhanced, while the binding ability of dephosphomimetic mutant Spastin QmA with GluA2 was impaired, when compared with the WT group. This phosphorylation-dependent interaction between Spastin and GluA2 was further confirmed by a Co-IP assay. As shown in Figure 1F, the interaction between Spastin QmA with GluA2 decreased significantly, whereas the opposite was seen for the binding ability of Spastin QmD to GluA2. Together, these results suggested that phosphorylation of Spastin increased its interaction with AMPAR GluA2.

### Phosphorylation of Spastin Increased the Surface Expression of AMPAR GluA2 and Synaptic Function

**Figure 2:**
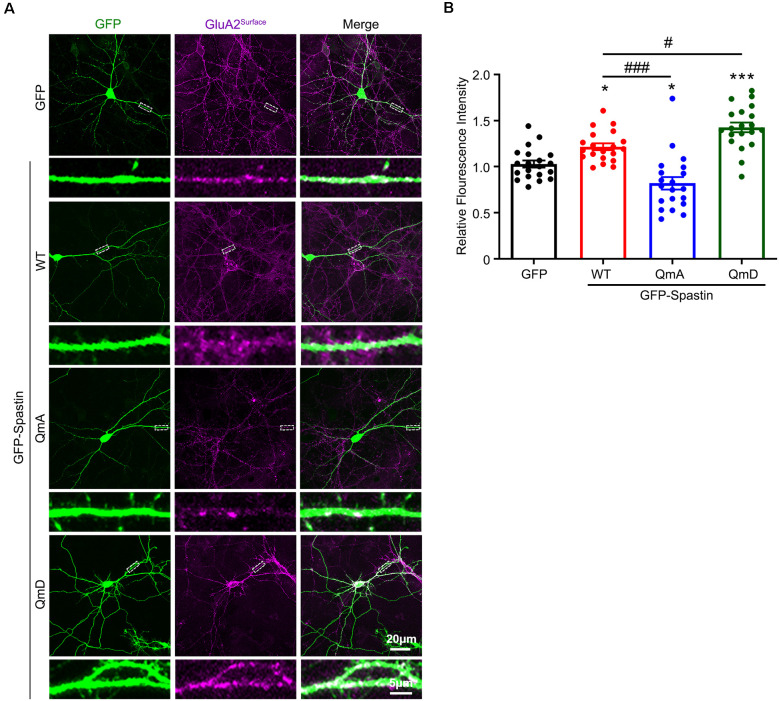
Phosphorylation of Spastin promoted GluA2 surface expression. **(A)** Confocal micrographs showing surface GluA2 in DIV 15 hippocampal neurons overexpressing GFP, GFP-Spastin WT, QmA, and QmD. Scale bar, 20 μm. The rectangle details were enlarged. In the magnified dendrite, the scale bar corresponds to, 5 μm. **(B)** Quantification of relative fluorescence intensity in neurons overexpressing GFP, GFP-Spastin WT, QmA, and QmD. In each group, *n* = 20 cells from three independent experiments, *P < 0.05, ***P < 0.001, as compared to the GFP group, ^#^*P* < 0.05, ^###^*P* < 0.001, as compared to the GFP-Spastin WT group.

To further clarify the effects of the interaction between Spastin and GluA2 on AMPAR levels, we monitored surface AMPAR levels *via* immunofluorescence staining of nonpermeabilized neurons with anti-GluA2 antibodies. As shown in Figure 2, neurons overexpression of Spastin exhibited an increasement of surface GluA2 fluorescence intensity compared with neurons overexpressing GFP. Additionally, neurons overexpressing Spastin QmA showed a decreased level of surface GluA2 fluorescence intensity, whether compared with neurons overexpressing GFP or Spastin WT. However, neurons overexpressing Spastin QmD exhibited an increased level in surface GluA2 fluorescence intensity when compared with neurons overexpressing GFP and Spastin WT. These data illustrated that Spastin phosphorylation increased the surface expression of AMPAR GluA2.

**Figure 3:**
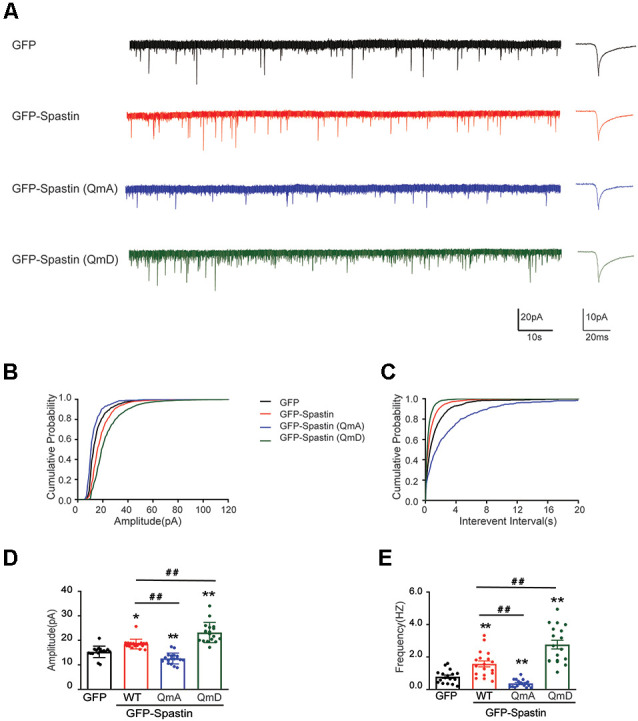
Phosphorylation of Spastin increased the amplitude and frequency of mEPSCs. **(A)** Representative recordings of mEPSCs from neurons overexpressing GFP, GFP-Spastin WT, QmA, and QmD. Representative traces of individual mEPSC are on the right. **(B,C)** Cumulative distributions of mEPSC amplitudes and the inter-mEPSC event intervals in neurons overexpressing GFP, GFP-Spastin WT, QmA, and QmD. **(D,E)** Quantification of mEPSCs amplitude and frequency in neurons overexpressing GFP, GFP-Spastin WT, QmA, and QmD. In GFP group *n* = 14 cells; in the WT, QmA and QmD group, *n* = 18 cells respectively; from three independent experiments, *P < 0.05, **P < 0.01, as compared to the GFP group, ^##^*P* < 0.01, as compared to the GFP-Spastin WT group.

Does the increased surface expression of AMPAR reflect in synaptic function? To determine whether the synaptic functional level of neurons overexpressing Spastin and its phosphorylation were changed, whole-cell patch clamp recordings were performed to measure mEPSCs levels in target neurons. As shown in Figure 3, neurons overexpressing Spastin WT exhibited an increased level in both amplitude and frequency of mEPSCs than the neurons overexpression GFP, indicating an increased number of functional synapses. Furthermore, compared with neurons overexpressing Spastin WT, neurons overexpressing Spastin QmD exhibited an increased level in amplitude and frequency of mEPSC, while neurons overexpressing Spastin QmA showed a decreased level in amplitude and frequency of mEPSC. All these data demonstrated that phosphorylation of Spastin increased the surface expression of AMPAR GluA2 and synaptic function.

### The Ser210 Site Phosphorylation of Spastin Contributed to the Surface Delivery of AMPAR

**Figure 4:**
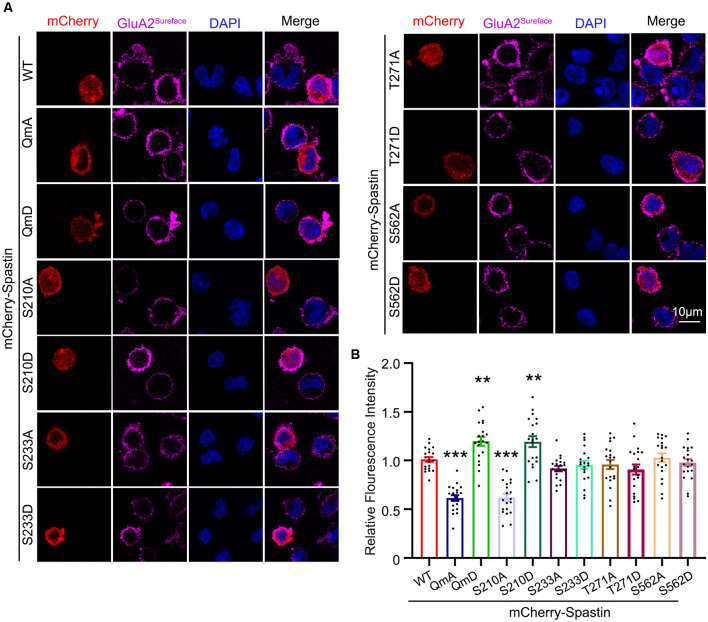
Phosphorylation of Spastin at Ser210 promoted GluA2 surface expression in HEK293T cells expressing GluA2 subunit. **(A)** Confocal micrographs showing surface GluA2 in HEK293T cells stably expressing the GluA2 subunit of AMPA receptor expressing mCherry-Spastin WT and its phosphomimetic and dephosphomimetic mutants, respectively. Scale bar, 10 μm. **(B)** Quantification of relative fluorescence intensity in HEK293 cells overexpressing mCherry-Spastin WT and its phosphomimetic and dephosphomimetic mutants, respectively. In each group, *n* = 20 cells from three independent experiments, **P < 0.01, ***P < 0.001, as compared to the mCherry-Spastin WT group.

**Figure 5:**
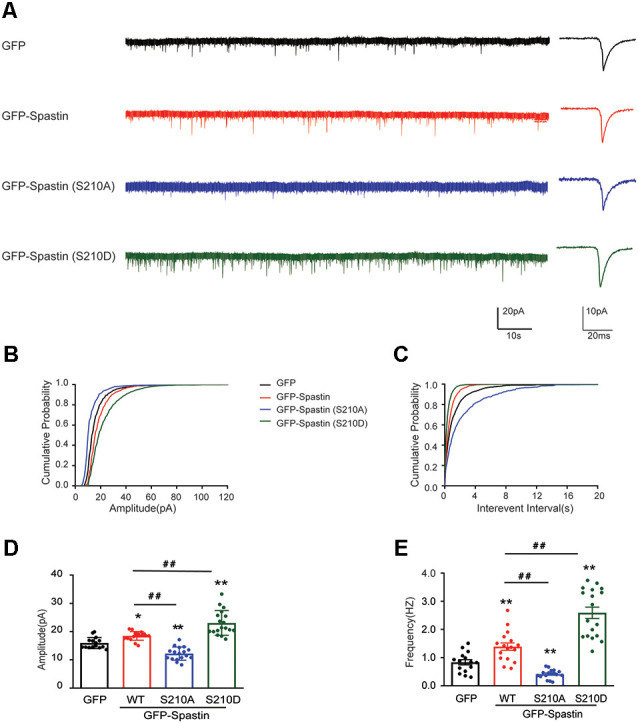
Phosphorylation of Spastin at Ser210 increased the amplitude and frequency of mEPSCs.** (A)** Representative recordings of mEPSCs from neurons overexpressing GFP, GFP-Spastin WT, S210A, and S210D. Representative traces of individual mEPSC are on the right. **(B,C)** Cumulative distributions of mEPSC amplitudes and the inter-mEPSC event intervals in neurons overexpressing GFP, GFP-Spastin WT, S210A, and S210D. **(D,E)** Quantification of mEPSCs amplitude and frequency in neurons overexpressing GFP, GFP-Spastin WT, S210A, and S210D. In GFP group *n* = 15 cells; in the WT and S210A group, *n* = 17 cells respectively; in QmD group, *n* = 18 cells; from three independent experiments, *P < 0.05, **P < 0.01, as compared to the GFP group, ^##^*P* < 0.01, as compared to the GFP-Spastin WT group.

After clarifying the relationship between Spastin phosphorylation and AMPAR transport, we asked which of the four phosphorylation sites played an important role during this process. To answer this question, we first generated a series of mutants with Ser210, Ser233, Thr271, and Ser562 replaced with alanine (S210A, S233A, T271A, and S562A) or aspartic acid (S210D, S233D, T271D, and S562D) to mimic dephosphorylated or phosphorylated Spastin, respectively. Next, we examined the level of GluA2 surface expression in HEK293T cells stably expressing the GluA2 subunit of AMPAR. HEK293T cells stably expressing the GluA subunits make it easier for us to observe the effects of AMPAR binding proteins on AMPAR trafficking (Wei et al., 2016; Li et al., 2018; Cheng et al., 2022). We found that cells transfected with Spastin S210D mutants, similarly to cells transfected with Spastin QmD mutants, displayed an increased surface expression of GluA2, whereas cells transfected with Spastin S210A mutants showed a reduced surface expression of GluA2 (Figure 4). To further explore the effect of Spastin S210 phosphorylation on excitatory synapses, whole-cell patch-clamp recordings were performed to analyze mEPSC of neurons transfected with Spastin WT, Spastin S210A, and Spastin S210D mutants. As shown in Figure 5, neurons overexpressing Spastin S210A showed a reduction in both amplitude and frequency of mEPSC, while neurons overexpressing Spastin S210D exhibited an increased level in amplitude and frequency of mEPSC, compared with neurons overexpressing GFP control. Moreover, the same phenomenon also appeared when compared with neurons overexpressing Spastin WT. Taken together, these results indicated that phosphorylation of Spastin at Ser210 alone may increase the surface expression of AMPAR GluA2.

### Phosphorylation of Spastin Promoted AMPAR Glua2 Surface Expression Independently of Microtubule Dynamics

**Figure 6:**
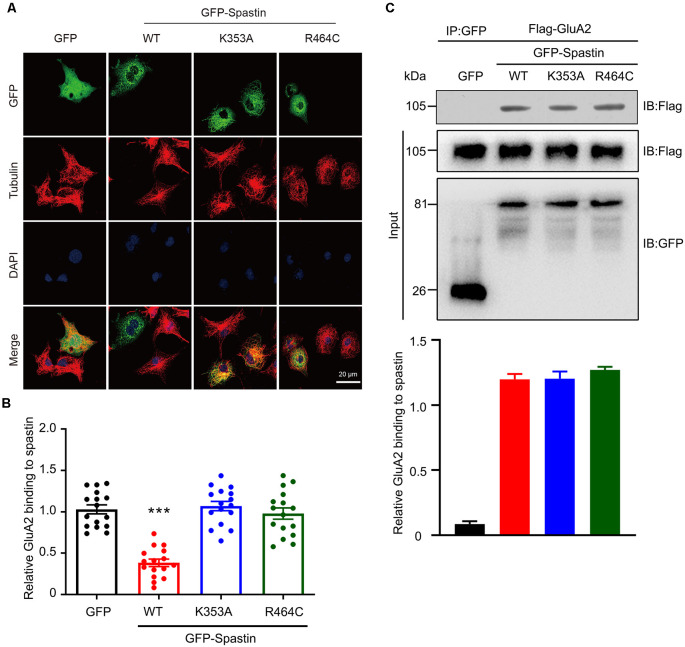
Mutation of Spastin at K353 and R464 inhibited microtubule-severing activity of Spastin but did not affect its binding to GluA2. **(A)** Confocal micrographs showing microtubules (red) in COS1 cells overexpressing GFP, GFP-Spastin WT, K353A, and R464C. Scale bar, 20 μm. **(B)** Quantification of relative fluorescence intensity of microtubules in cells overexpressing GFP, GFP-Spastin, and its mutants. In each group, *n* = 16 cells from three independent experiments, ***P < 0.001 compared to the GFP group. **(C)** HEK293T cells co-transfected with Flag-GluA2 and GFP, GFP-Spastin, GFP-Spastin K353A, and GFP-Spastin R464C were lysed for Co-IP assay. Immunoblotting of input and bound proteins was performed using antibodies against GFP and Flag, respectively (top). Quantification of the relative binding of GluA2 to GFP, GFP-Spastin, GFP-Spastin K353A, and GFP-Spastin R464C (bottom), *n* = 3 independent experiments.

The classical function of Spastin is to cut microtubules and we recently reported that phosphorylation of Spastin decreased the severing function of microtubules (Li et al., 2021). To determine whether the increased level of surface GluA2 is associated with their binding to Spastin or with the microtubule-severing efficiency of Spastin, we first generated two mutants of Spastin without the ability to sever microtubules: Spastin K353A (mutation of lysine to alanine) and Spastin R464C (mutation of arginine to cysteine) according to a previous study (Evans et al., 2005). Then, these two mutants were transfected into COS1 cells to verify their microtubule-severing function. Microtubule immunofluorescence staining showed that the microtubules in the COS1 cells transfected with the Spastin WT were severed into microtubule segments or fragments when compared with the cells transfected with GFP control, while cells overexpressing Spastin K353A or Spastin R464C had intact microtubules retained (Figure 6A). The quantitative analysis showed that the relative microtubules’ fluorescence intensity decreased significantly in cells overexpressing Spastin WT when compared with those in control cells overexpressing GFP. However, there was no difference in microtubule fluorescence intensity, among cells overexpressing K353A and R464C and control cells overexpressing GFP (Figure 6B). These data indicated that K353A and R464C obliterated the ability to sever microtubules, which is consistent with previous research (Evans et al., 2005).

In addition, we also evaluated if the interaction between GluA2 and Spastin would be affected by the two mutants using Co-IP assays. We found that Spastin WT, Spastin K353A, and Spastin R464C coimmunoprecipitated almost an equal amount of GluA2 (Figure 6C). These data suggested that mutation of Spastin at K353 and R464 did not affect the interaction between Spastin and GluA2.

**Figure 7:**
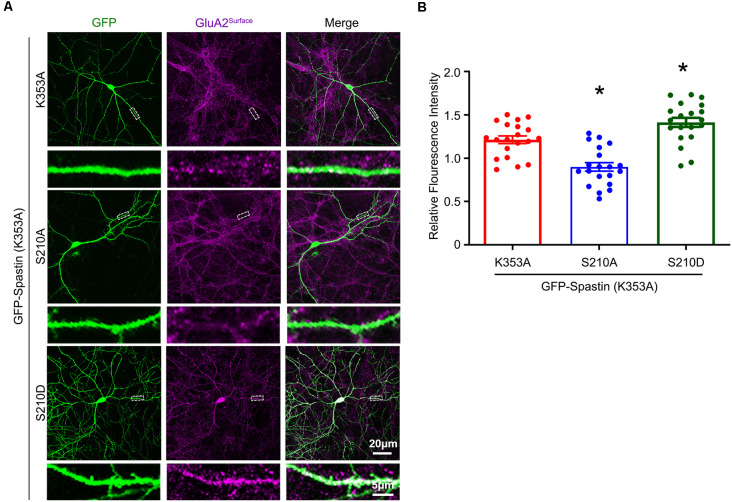
Phosphorylation of Spastin at Ser210 with impaired microtubule-severing activity promoted GluA2 surface expression. **(A)** Confocal micrographs showing surface GluA2 in DIV 15 hippocampal neurons overexpressing GFP-Spastin K353A, GFP-Spastin K353A/S210A, and GFP-Spastin K353A/S210D. Scale bar, 20 μm. The rectangle details were enlarged. In the magnified dendrite, the scale bar corresponds to 5 μm. **(B)** Quantification of relative fluorescence intensity in neurons overexpressing GFP-Spastin K353A, GFP-Spastin K353A/S210A, and GFP-Spastin K353A/S210D. In each group, *n* = 20 cells from three independent experiments, *P < 0.05, as compared to the GFP-Spastin K353A group.

**Figure 8:**
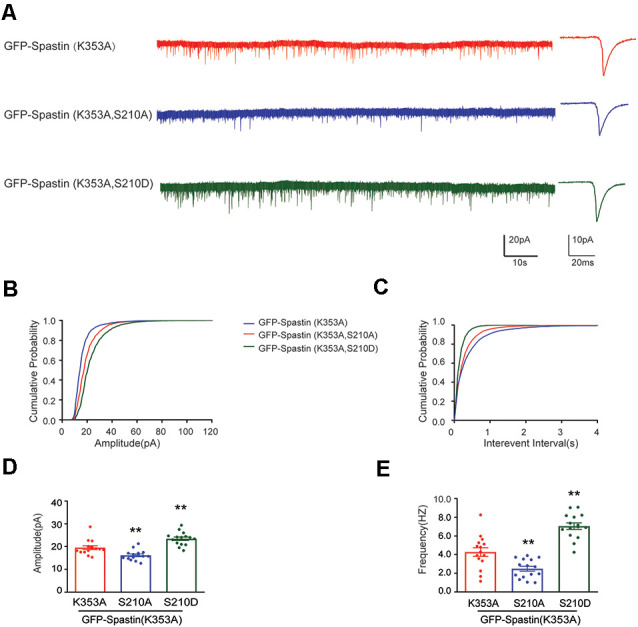
Phosphorylation Spastin at Ser210 with microtubule-severing activity impaired increased the amplitude and frequency of mEPSCs. **(A)** Representative recordings of mEPSCs from neurons overexpressing GFP-Spastin K353A, GFP-Spastin K353A/S210A and GFP-Spastin K353A/S210D. Representative traces of individual mEPSC are on the right. **(B,C)** Cumulative distributions of mEPSC amplitudes and the inter-mEPSC event intervals in neurons overexpressing GFP-Spastin K353A, GFP-Spastin K353A/S210A, and GFP-Spastin K353A/S210D. **(D,E)** Quantification of mEPSCs amplitude and frequency in neurons overexpressing GFP-Spastin K353A, GFP-Spastin K353A/S210A, and GFP-Spastin K353A/S210D. In each group, *n* = 15 cells from three independent experiments, **P < 0.01, as compared to the GFP-Spastin K353A group.

After clarifying that Spastin K353A and R464C can lose the microtubule-severing function without changing the binding ability between Spastin and GluA2, Spastin phosphomimetic and dephosphomimetic mutants with impaired microtubule-severing activity (K353A/S210A and K353A/S210D) were generated. Then, immunofluorescence staining and electrophysiology assessments were performed to explore the effect of Spastin S210 phosphorylation on the trafficking of AMPAR after Spastin K353A, Spastin K353A/S210A, and Spastin K353A/S210D were transfected into the hippocampal neurons. As shown in Figure 7, neurons overexpressing Spastin K353A/S210A showed a decreased surface GluA2 fluorescence intensity compared with neurons overexpressing Spastin K353A. However, neurons overexpressing Spastin K353A/S210D exhibited increased surface GluA2 fluorescence intensity when compared with neurons overexpressing Spastin K353A. Simultaneously, electrophysiological findings revealed that neurons overexpressing Spastin K353A/S210A exhibited a reduction in both amplitude and frequency of mEPSC compared with neurons overexpressing Spastin K353A. In contrast, neurons overexpressing Spastin K353A/S210D exhibited an increased level in amplitude and frequency of mEPSC when compared with neurons overexpressing Spastin K353A (Figure 8). These data indicated that phosphorylation of Spastin at Ser210 promoted AMPAR GluA2 surface expression independent of microtubule dynamics.

## Discussion

The present study demonstrated that Spastin interacted with AMPAR and phosphorylation of Spastin enhanced its interaction with AMPAR subunit GluA2. Further immunostaining and electrophysiology experiments showed that phosphorylation of Spastin increased the surface expression of AMPAR GluA2 subunits and synaptic function. Additionally, our study further clarified that it was the Ser210 site phosphorylation of Spastin that contributed to the surface delivery of AMPAR and this process was not dependent on microtubule dynamics.

First, our results provide evidence that Spastin acts as a regulatory protein on postsynaptic AMPA receptors. In our previous study, we reported that Spastin promotes dendrite outgrowth (Ji et al., 2018). In this research, the results showed that overexpression of Spastin increased surface expression of AMPA receptors (Figure 2). Meanwhile, the synaptic function had also been strengthened as reflected by the amplitude and frequency of mEPSC (Figure 3). Increased amplitude and frequency of mEPSC indicate the presence of an increased number of AMPARs at the synapse. Interestingly, phosphorylation of Spastin further increased AMPAR surface expression and synaptic function, whereas dephosphorylated Spastin had the opposite effect. Here, Spastin showed the ability to transfer AMPARs to the membrane. This was especially true in the case of phosphorylated Spastin.

Second, one of the most striking results obtained from our research is the phosphorylation-dependent interaction between Spastin and AMPAR GluA2 subunit. Studies have shown that AMPAR transport involves a complex protein-protein interaction network. In our study, Spastin showed a degree of association with all the four subunits of AMPARs (Figure 1B). Although Spastin binding to AMPA receptors did not exhibit subunit specificity, it suggested that Spastin was an AMPAR binding protein. Due to the predominance of GluA2-containing AMPARs in hippocampal neurons (Bats et al., 2013), we focused on investigating the binding ability between Spastin and GluA2. Pulldown and Co-IP assays provided direct evidence that Spastin interacted with GluA2 and that their interaction was regulated by Spastin phosphorylation (Figures 1E,F). Protein phosphorylation, the most studied post-translational modification, is employed by cells to transiently alter protein properties such as their localization and conformation, as well as their interactions with other proteins (Sharma et al., 2014). Our recently published article also reported a phosphorylation-dependent binding of Spastin with tubulin. Phosphorylated Spastin weakens its binding to microtubules, thereby its microtubule-severing ability is attenuated (Li et al., 2021). On the contrary, here, phosphorylated Spastin showed increased binding ability with GluA2. This means that phosphorylated Spastin is dissociated from the microtubules to assist receptor transport.

The number of synaptic AMPARs is regulated through endocytosis, exocytosis, and endosomal sorting, which results in the recycling of AMPARs back to the plasma membrane or degradation in the lysosome (van der Sluijs and Hoogenraad, 2011; Parkinson and Hanley, 2018). Here, we confirmed that phosphorylated Spastin promotes receptor expression on the membrane by increasing its binding ability to GluA2, however, the specific step underlying the AMPARs Spastin trafficking pathway remains unclear. Previous studies have reported that Spastin interacts with IST1 and CHMP1B, the ESCRT-III-associated proteins, to control endosome recycling (Campsteijn et al., 2016; Connell et al., 2020). The process of AMPA receptors trafficking is dependent on endosomal recycling (van der Sluijs and Hoogenraad, 2011). It is therefore hypothesized that Spastin may serve as an intermediate protein to interact with AMPAR subunits in hippocampal neurons, thereby affecting the entry and exit of AMPAR subunits to the membrane, and positively regulating the synaptic function. In addition, Spastin QmA decreased synaptic function and AMPAR surface expression even more than GFP, which was not anticipated. We speculate that the site mutation may have a greater impact on the conformation of non-phosphorylated spastin. On one hand, dephosphorylation greatly reduces the recruitment of spastin to AMPA receptors, and on the other hand, it may also affect the binding of proteins related to endosome recycling. However, these speculations require further investigation.

Third, our results identified the key phosphorylation site of Spastin involved in AMPAR trafficking. There are four different potential phosphorylation sites in Spastin. In our study, we started by mutating all four Spastin phosphorylation sites to construct phosphomimetic (Spastin QmD) and dephosphomimetic mutants (Spastin QmA) of Spastin. After clarifying the role of Spastin phosphorylation in AMPAR transportation, we generated a series of mutants, with Ser210, Ser233, Thr271, and Ser562 replaced with alanine (S210A, S233A, T271A, and S562A) or aspartic acid (S210D, S233D, T271D, and S562D), respectively, to mimic dephosphorylated or phosphorylated Spastin and determine which site played a key role in this process. We found that like the “quadruple phosphomutants,” the “single phosphomutants” at S210 of Spastin could promote the GluA2 surface expression and the amplitude and frequency of mEPSCs. Other phosphorylation sites of Spastin did not show any effect. Thus, S210 phosphorylation appears to be crucial for Spastin in AMPAR trafficking. Simultaneously, S210 phosphorylation also has been reported to play a role in neurite outgrowth and branching (Li et al., 2021). In addition, HIPK2 phosphorylates Spastin at S268 contributes to midbody localization for successful abscission in cytokinesis (Pisciottani et al., 2019). This suggests that Spastin regulates different biological functions through phosphorylation at different sites. However, with the exception of S268, which is reported to be phosphorylated by HIPK2, the upstream kinase at other sites is still unknown. Thus, investigating the role and the upstream kinase of other phosphorylation sites will be an interesting topic for future research.

Finally, our study clarified whether microtubule motility was involved in Spastin phosphorylation-mediated AMPAR trafficking. Previous studies have revealed that loss of Spastin can cause abnormalities in the stability of the neuronal microtubule cytoskeleton and lead to synaptic growth and neurotransmission defects (Trotta et al., 2004; Ji et al., 2018). Through its MTBD domain, Spastin binds to the microtubule, then the fence-like structure of microtubules is disrupted by its AAA ATPase domain. Thus, long microtubules are severed into small microtubule fragments by Spastin (Garnham and Roll-Mecak, 2012). Moreover, in our previous study, we reported that phosphorylation of Spastin decreased the microtubule-severing ability (Li et al., 2021). Therefore, we needed to clarify whether the surface expression of GluA2 increased by Spastin phosphorylation is related to the dynamics of microtubules. In our present study, we generated two Spastin mutants, which obliterated the microtubule-severing function. Subsequently, we confirmed that their microtubule-severing function was indeed impaired and that their interaction with GluA2 was not changed by immunofluorescence staining of microtubules and by Co-IP assay, respectively (Figure 6). Therefore, Spastin phosphomimetic and dephosphomimetic mutants with impaired microtubule-severing activity (K353A/S210A and K353A/S210D) were produced as a tool for studying the effect of Spastin S210 phosphorylation on AMPAR trafficking. In the case that Spastin obliterated its microtubule cleavage activity, phosphorylation of Spastin at S210 could still promote the GluA2 surface expression and improve the frequency and amplitude of mEPSCs (Figures 7, 8). Thus, a pivotal role of Spastin S210 phosphorylation in the trafficking of AMPARs was confirmed, and this process was independent of microtubule dynamics. Therefore, it is plausible to conclude that the binding ability between Spastin and GluA2 is critical for AMPAR delivery to neuronal membranes.

Mutations in *SPAST* are the most common cause of HSP (Kara et al., 2016; Erfanian Omidvar et al., 2021). Most research on Spastin has focused on its relationship with movement disorders of HSP. Although increasing research has shown that SPG4-linked HSP is associated with cognitive dysfunction (Orlacchio et al., 2004; Murphy et al., 2009; Chelban et al., 2017; Akaba et al., 2021; Erfanian Omidvar et al., 2021; Giordani et al., 2021), the studies on its mechanism remain few. Brain abnormalities in regions including the cerebral cortex, corpus callosum, hippocampus, and thalamus were observed in patients with SPG4-HSP (Orlacchio et al., 2004; Murphy et al., 2009; Servelhere et al., 2021). In addition, mice with spastin depletion exhibited working and associative memory deficits and reduced function of hippocampal synapses (Lopes et al., 2020). These studies suggest that the function of spastin is closely related to synaptic plasticity and cognitive function. Thus, we chose to use cultured hippocampal neurons to study the relationship between spastin and AMPA receptors. Our study primarily, demonstrated that phosphorylation of Spastin promotes the surface delivery and synaptic function of AMPA receptors; however, whether this will cause changes in synaptic plasticity and cognitive functions needs to be further confirmed *in vivo*. In addition, since the upstream kinase of Spastin S210 phosphorylation is unknown, we relied on overexpression of various constructs in primary hippocampal neurons. Pharmacological manipulations will shed light on this physiological process once the upstream kinases are identified. In addition, our previous studies have confirmed spastin phosphorylation at site S210 in rat brain tissue, although it is unclear whether the Spastin S210 phosphorylation level is reduced in the brain tissue of patients with SPG4-HSP. Nevertheless, Spastin phosphorylation is beneficial for enhancing AMPA receptor function, which may be a potential target for improving cognitive dysfunction in patients with SPG4-HSP in the future.

In summary, this study reveals a novel role of Spastin on AMPAR trafficking. It provides the first evidence that Spastin interacts with AMPAR and demonstrated that their phosphorylation-dependent interactions rather than microtubule dynamics are required for GluA2 surface delivery. Finally, we also verified that it is Ser210 site phosphorylation of Spastin that contributed to GluA2 AMPAR trafficking. Our study provides important evidence on the role of Spastin phosphorylation in AMPAR trafficking, which will advance our understanding of cognitive dysfunction in HSP.

## Data Availability Statement

The original contributions presented in the study are included in the article, further inquiries can be directed to the corresponding author/s.

## Ethics Statement

The animal study was reviewed and approved by Institutional Animal Care and Use Committee at Jinan University.

## Author Contributions

JifZ and GG conceived and designed the study. JifZ wrote the article. LC, HW, and SC performed most of the experiments and analyzed the data. JL, JiaZ, and JW helped with pulldown and Co-IP experiments. All authors contributed to the article and approved the submitted version.

## Conflict of Interest

The authors declare that the research was conducted in the absence of any commercial or financial relationships that could be construed as a potential conflict of interest.

## Publisher’s Note

All claims expressed in this article are solely those of the authors and do not necessarily represent those of their affiliated organizations, or those of the publisher, the editors and the reviewers. Any product that may be evaluated in this article, or claim that may be made by its manufacturer, is not guaranteed or endorsed by the publisher.

## Footnotes

^1^ https://www.phosphosite.org
